# Electrosynthesis of Silver Particles–Polypyrrole on Screen-Printed Carbon Electrodes, with a View to Their Modification with Ki-67 Antibodies

**DOI:** 10.3390/polym18080909

**Published:** 2026-04-08

**Authors:** Matias Luengo, Loreto A. Hernández, Isabeau D. M. Figueroa, Cindy Peña, Gonzalo Riveros, Eduardo Muñoz

**Affiliations:** 1 Facultad de Ciencias, Instituto de Química y Bioquímica, Universidad de Valparaíso, Valparaíso 2362735, Chile; matias.luengo@postgrado.uv.cl (M.L.); isabeau.fj@gmail.com (I.D.M.F.); gonzalo.riveros@uv.cl (G.R.); 2 Preclinical Department, Medicine School, Universidad de Valparaíso, Valparaíso 2362735, Chile; cindy.pena@uv.cl; 3 Facultad de Ciencias, Instituto de Química, Pontificia Universidad Católica de Valparaíso, Valparaíso 2373223, Chile

**Keywords:** biosensor, PPy, AgPs, Ki-67

## Abstract

The development of reliable electrochemical interfaces for biosensor applications requires materials that combine high conductivity, large effective surface area, and suitable platforms for biomolecule immobilization. In this work, a hybrid electrochemical platform based on screen-printed carbon electrodes (SPCEs) modified with electropolymerized polypyrrole (PPy) and electrodeposited silver particles (AgPs) is presented for the subsequent immobilization of Ki-67 antibodies. PPy films were synthesized under optimized electrochemical conditions, producing homogeneous, porous, and electrochemically stable coatings that significantly enhanced the doping/undoping processes from 0.3280 C/0.3284 C to 0.3281 C/0.3284 C for SPCE and SPCE-PPy, respectively. Subsequently, silver particles were deposited onto the PPy matrix, resulting in a well-dispersed and uniform distribution of AgPs, promoted by the interaction between Ag^0^ and the nitrogen groups in the polymer backbone. The synergistic combination of PPy and AgPs resulted in improved charge-transfer properties and enhanced electrochemical reversibility, thereby decreasing the peak-to-peak separation of the ferricyanide/ferrocyanide redox couple used as a probe by 40%. Immobilization of Ki-67 antibodies was achieved via direct interaction with AgPs, resulting in a marked passivation effect, as evidenced by the suppression of redox probe signals, confirming successful biofunctionalization. The proposed SPCE-PPy-AgP architecture provides a robust, reproducible, and versatile platform for antibody immobilization, as demonstrated by oxidation and reduction peaks with relative standard deviations (RSDs) of 3.18% and 4.43%, respectively, highlighting its potential for developing label-free electrochemical immunosensors for clinically relevant proliferation biomarkers.

## 1. Introduction

The development of sensitive, selective, and reproducible analytical platforms for detecting clinically and/or analytically relevant species in environmental monitoring remains a major challenge in the field of electrochemical biosensors [1,2,3]. In this context, biomarkers associated with cellular proliferation have attracted particular interest due to their applications in the diagnosis, prognosis, and monitoring of various pathologies, especially in oncology. Among these markers, the nuclear antigen Ki-67 is widely used to assess the proliferative fraction of tumor cells, as its expression is strictly associated with the active phases of the cell cycle and is absent in quiescent cells. This characteristic makes Ki-67 an attractive target for the development of specific and reliable detection systems that can provide relevant information in clinical and biomedical research settings.

Traditionally, Ki-67 detection is performed using immunohistochemical or immunofluorescence techniques [4,5]. Although these methods are highly specific and require specialized equipment, multiple sample-preparation steps, and trained personnel, they limit their use in decentralized or rapid diagnostic settings. In response to these limitations, electrochemical biosensors have emerged as a promising alternative, offering significant advantages, e.g., low cost, rapid analysis, miniaturization, and the potential for integration into portable devices. Thus, electrochemical immunosensors that immobilize specific antibodies on conductive surfaces have demonstrated effectiveness for detecting protein biomarkers, combining the selectivity of biological recognition with the sensitivity of electrochemical techniques.

Electrochemical biosensor performance is strongly influenced by the architecture of the electrode–solution interface, which must simultaneously fulfill functions related to signal transduction, biomolecule immobilization, and signal amplification [1,3,6,7]. Accordingly, conducting polymers have been extensively studied as materials for surface modification due to their high conductivity, electrochemical stability, and structural versatility. Among them, polypyrrole (PPy) [8,9,10,11,12] stands out because of its ease of electrochemical synthesis, biocompatibility, and ability to form porous films with a high effective surface area. These characteristics make PPy particularly suitable for biosensor development, as they facilitate both charge transfer and biomolecular immobilization.

The electropolymerization of PPy allows precise control over film thickness, morphology, and doping degree, parameters that directly influence its electrochemical properties. PPy films obtained by electrochemical techniques typically exhibit nodular or granular morphologies characterized by high surface roughness, three-dimensional porous structures, thereby significantly increasing the electroctrochemical active surface area. This porous matrix not only enhances electrochemical response but also provides an appropriate support for species such as metal nanoparticles, enabling the design of hybrid platforms with synergistic properties. Indeed, metal particles have been widely employed in biosensors due to their high conductivity, large surface-to-volume ratio, and ability to interact with biomolecules. In particular, the physicochemical properties of silver particles (AgPs) [13,14,15], e.g., excellent electrical conductivity and chemical affinity toward functional groups present in proteins, such as amino and thiol groups, enable direct antibody immobilization without complex chemical functionalization, simplifying biosensor design and reducing potential sources of variability.

Several studies have demonstrated that incorporating silver particles into conducting polymer matrices significantly improves charge transfer and electrochemical reversibility. Furthermore, the presence of a polymer such as PPy can modulate the nucleation and growth of metal particles, leading to a more homogeneous distribution and uniform particle size compared with deposits formed directly on bare electrodes. This feature is particularly relevant in biosensor applications, as controlled nanoparticle distribution improves electrode-to-electrode reproducibility and yields more consistent analytical responses.

Compared to other systems reported in the literature based on carbon matrices or metal oxides, hybrid PPy–metal platforms offer clear advantages in terms of conductivity, ease of fabrication, and compatibility with conventional electrochemical techniques [1,2]. Additionally, the use of screen-printed carbon electrodes (SPCEs) as substrates offers further benefits, including low cost, mass production capability, portability, and potential integration into disposable devices, which are key attributes for in situ diagnostic applications.

In this work, an electropolymerized PPy film combined with electrodeposited Ag particles on SPCEs was developed as an active electrochemical platform for the immobilization of the Ki-67 antibody and the sensitive transduction of biomolecular recognition events. Accordingly, this study focuses on the development and characterization of the SPCE–PPy–AgNP interface, evaluating its electrochemical and morphological properties at each modification step. The results demonstrate that this system provides a suitable basis for the future development of label-free electrochemical immunosensors. Moreover, the versatility of this approach suggests that it may be extended to the detection of other clinically relevant biomarkers and environmentally relevant analytes, highlighting hybrid platforms based on conducting polymers and metallic nanoparticles as promising tools for electrochemical biosensors.

## 2. Materials and Methods

All experiments were performed at room temperature (20 °C) under an inert argon atmosphere in a three-compartment beaker glass cell. Screen-printed carbon electrodes (SPCEs) with a geometric area of 1 cm^2^ were used as the working electrodes, which contain a carbon auxiliary electrode and an Ag/AgCl electrode employed as the reference electrode. All potentials reported in this work are referenced to this electrode.

Polypyrrole (PPy) (Aldrich, 98%) films were grown by cyclic voltammetry in a potential ranging from 0.0 to 1.4 V at a scan rate of 0.1 V s^−1^ using solutions containing 0.01 M of pyrrole and 0.1 M of tetrabutylammonium hexafluorophosphate (TBAPF_6_) as support electrolyte (Aldrich, 98%) in acetonitrile (CH_3_CN) (Merck, gradient grade for liquid chromatography). Silver nanoparticles (AgPs) were subsequently deposited by chronoamperometry by applying a potential of −0.50 V for 3 s from an aqueous solution containing 0.05 M of Ag_2_SO_4_ (Aldrich, 99.9%) and 2.3 M of KSCN (Merck, Rahway, NJ, USA) prepared in ultrapure Milli-Q water.

For the preliminary Ki-67 antibody immobilization assay, one modified electrode was incubated with a Ki-67 antibody solution (10 µg mL^−1^) for 1 h at room temperature, followed by a 15 min wash in phosphate-buffered saline (PBS) at pH 7.3 to remove unbound antibodies. Finally, the electrode was carefully rinsed with Milli-Q water to remove traces of PBS before electrochemical testing. To test the stability of the platform, CV was performed right after preparation and 24 h post-preparation to compare the profiles. For interference testing, a ferricyanide solution was prepared with 5 mM of glucose or ascorbic acid before testing the CV response of the modified electrodes.

All electrochemical measurements were carried out using a CH Instruments CHI660D potentiostat (CH Instruments, Inc., Bee Cave, TX, USA). Morphological characterization of the samples was performed using a field-emission scanning electron microscope (FESEM Quattro S, Thermo Fisher Scientific, Waltham, MA, USA).

## 3. Results and Discussion

### 3.1. Electrosynthesis and Characterization of SPCE–PPy

To obtain PPy films, a 0.01 M pyrrole (Py) solution containing 0.1 M TBAPF_6_ as the supporting electrolyte in CH_3_CN was employed, using direct electrosynthesis onto SPCEs. This electrochemical process can be described by the following equation [16]:

(1) $$(n+2)HPyH_{\left(aq\right)}\to HPy{\left(Py\right)}_{n}PyH_{\left(s\right)}^{\left(nx\right)}+(2n+2)H_{\left(aq\right)}^{+}+(2n+2+nx)e^{-}$$

In this reaction, Py is oxidized, forming doped PPy and protons and electrons. In this equation, *x* represents the degree of doping of the polymer, typically ranging from 0.25 to 0.33 [2,3].

**Figure 1:**
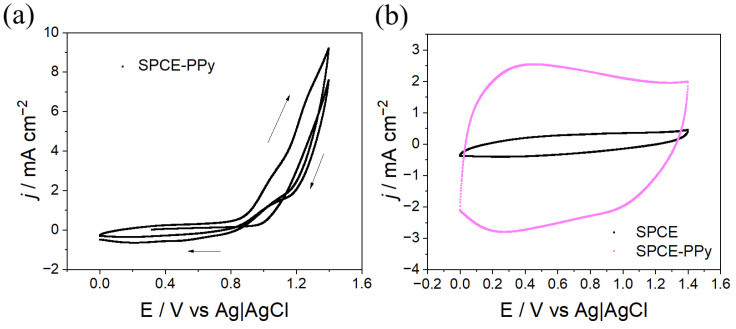
(**a**) Cyclic voltammogram for the electrosynthesis of PPy on SPCE. Three cycles, v = 100 mV s^−1^. Py 0.1 M + TBAPF_6_ 0.1 M in CH_3_CN. (**b**) Electrochemical response of SPCE and SPCE–PPy in 0.1 M TBAPF_6_ in CH_3_CN, v = 100 mV s^−1^.

In a first stage, the parameters required for the electrosynthesis of PPy on SPCEs were investigated by cyclic voltammetry (CV). It was found that the polymer grows within a potential window from 0 V to 1.4 V at a scan rate of 50 mV s^−1^ (Figure 1a). As observed between 1.0 V and 1.4 V, the current increases with each successive cycle, indicating polymer growth on the electrode surface.

After synthesis, homogeneous PPy films with a dark, opaque blue color were obtained on the SPCE surface. The electrochemical responses of the synthesized polymer, corresponding to the charging and discharging processes, were recorded using a solution composition identical to that employed for polymerization in the absence of monomer (0.1 M TBAPF_6_ in CH_3_CN). Measurements were performed until signal stabilization, and the final cycle was plotted (Figure 1b). This procedure allows extraction of electrochemical parameters, confirming that PPy is not overoxidized during polymerization within the employed potential windows, as the responses stabilize and consecutive cycles overlap without film degradation. This behavior suggests that the PPy coatings exhibit good electrochemical stability, making them suitable for subsequent surface modifications. Additionally, as shown in Figure 1b, the charge–discharge processes of PPy are markedly enhanced compared to those observed for the bare SPCE. In fact, when analyzing the doping/undoping processes by comparing SPCE and SPCE-PPy, significant improvements in the obtained charge values are observed, as shown in Table 1.

**Figure 2:**
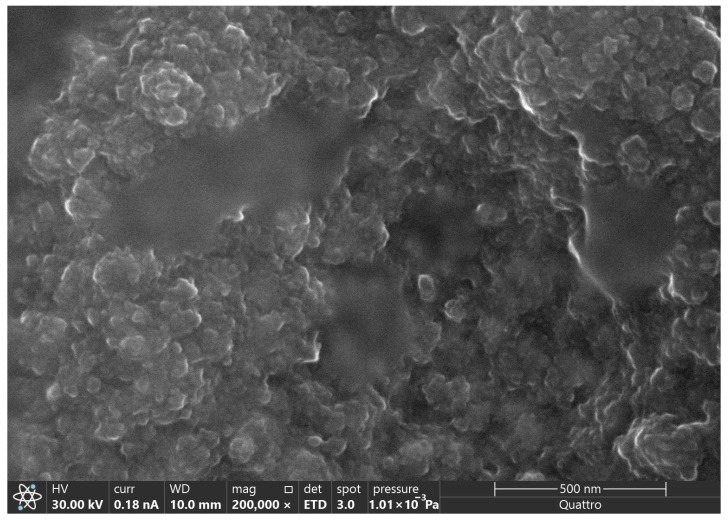
FESEM micrograph of PPy obtained by CV.

The surface morphology of the PPy film was examined by SEM and is shown in Figure 2. The micrographs reveal the formation of a rough, heterogeneous coating with a granular, nodular morphology. The PPy layer deposited on SPCE consists mainly of globular aggregates that coalesce to form a typical “cauliflower-like” structure, commonly reported for electropolymerized conducting polymers. These closely packed nodules, of varying sizes and shapes, exhibit a highly textured, porous structure.

The high surface roughness and porosity observed are advantageous, as they increase the effective surface area and provide an appropriate matrix for incorporating functional species, i.e., Ag particles. Overall, the SEM analysis confirms that electrochemical synthesis yields a well-adhered PPy film with adequate porosity and increased surface area.

**Figure 3:**
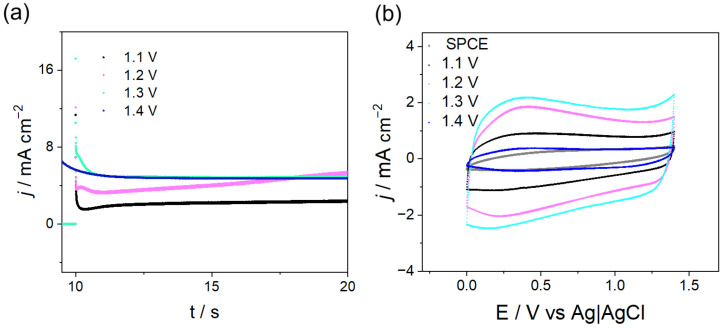
(**a**) Current density–time (j/t) transients for the electrosynthesis of SPCE–PPy using 10 s pulses at different applied potentials (Py 0.1 M + TBAPF_6_ 0.1 M in CH_3_CN). (**b**) Electrochemical response of (**a**) (v = 100 mV s^−1^ in 0.1 M TBAPF_6_ in CH_3_CN).

The optimization of PPy synthesis was performed by chronoamperometry (CA) at potentials where an exponential increase in current was observed in CV (1.1–1.4 V, Figure 1). The corresponding results are shown in Figure 3. Using appropriate pulse times enabled homogeneous coating formation and electrochemical responses comparable to those obtained by CV. For all the applied potentials (Figure 3a), the recorded current transients follow a behavior reported in the literature for this polymer [4,5], i.e., the formation of the electrical double layer at the electrode interface, followed by the nucleation and growth of the first PPy nuclei on the surface. In addition, it was observed that applying a potential of 1.3 V resulted in a transient with sustained current growth, which correlates with a larger mass of PPy deposited. When this potential pulse was applied for 10 s, complete coverage of the SPCE surface was achieved.

Subsequently, the electrochemical responses of these deposits were evaluated using CV (Figure 3b). The CV response obtained for the film formed at a pulse width of 10 s, and a potential of 1.3 V shows a higher signal, attributed to an increase in the charge-transfer product of the effective electrode area, confirming that a larger mass of polymer is deposited at this potential [6,7]. Potentials below 1.3 V could indicate insufficient chain growth for polymer precipitation onto the electrode surface, whereas potentials above 1.3 V may lead to overoxidation of the film, resulting in electrochemically and environmentally unstable deposits, as evidenced by the corresponding electrochemical responses.

**Figure 4:**
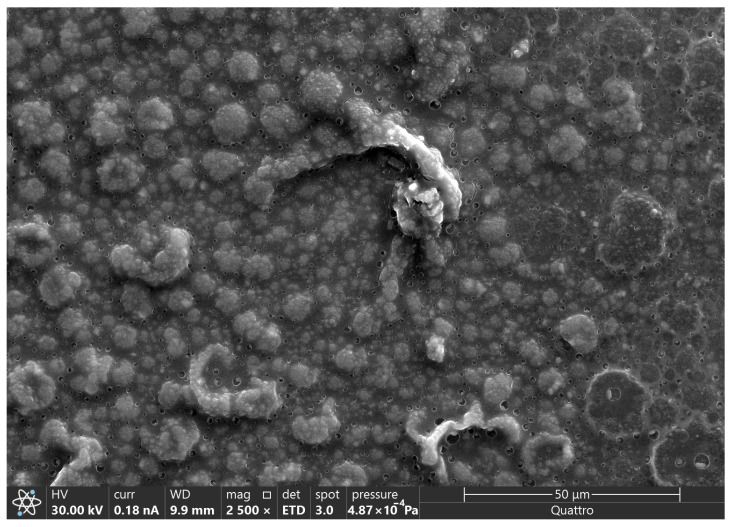
FESEM micrograph of PPy deposited on FTO obtained by chronoamperometry (1.3 V pulse).

Figure 4 shows FESEM images of the electrode surface, which confirms the presence of PPy, as evidenced by its characteristic rough morphology [8,9,10,11,12]. In addition, the presence of interstitial voids between adjacent nodules suggests a microporous network, likely formed by nucleation and growth during electrochemical polymerization. This morphology is consistent with a progressive nucleation 3D with a diffusional-controlling growth on the electrode surface.

### 3.2. Electrochemical Preparation of SPCE–AgPs

To optimize the electrosynthesis of Ag on SPCE–PPy, preliminary exploratory experiments were first carried out on bare SPCEs, following the procedure reported by Riveros et al. [13]. This reaction proceeds through two consecutive stages, which can be described as follow:

(2) $$KSCN_{\left(aq\right)}+{Ag}_{2}{SO}_{4\left(aq\right)}\to Ag{{\left(SCN\right)}_{3}^{-2}}_{\left(aq\right)}$$

(3) $$Ag{{\left(SCN\right)}_{3}^{-2}}_{\left(aq\right)}+\;e^{-}\to {Ag}_{\left(s\right)}^{0}+3{SCN}_{\left(aq\right)}^{-}$$

According to Equation (2), in a first stage, the Ag(SCN)_3_^2−^ complex is formed, which is subsequently reduced in a second electrochemical step to produce metallic Ag (Equation (3)).

**Figure 5:**
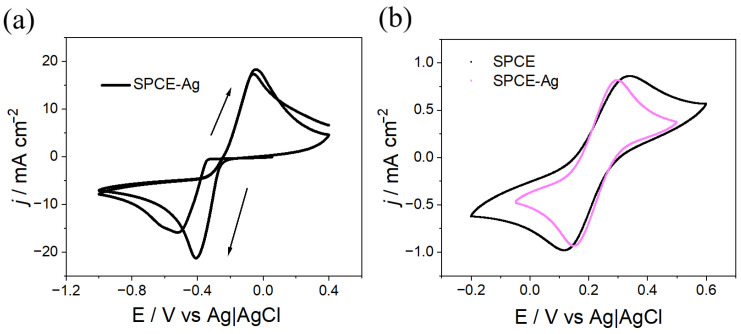
(**a**) Cyclic voltammogram for the electrosynthesis of SPCE–Ag (0.05 M Ag_2_SO_4_ + 2.3 M KSCN in Milli-Q water, v = 100 mV s^−1^, two cycles). (**b**) Electrochemical response of SPCE and SPCE–Ag (5 mM K_3_[Fe(CN)_6_] + 0.1 M KCl in Milli-Q water, v = 100 mV s^−1^).

As shown in Figure 5a, during the first CV cycle a reduction peak appears at −0.61 V, while during the second cycle a peak is observed at −0.49 V. This behavior arises because, during the first cycle, stable nuclei are formed on the electrode surface, giving rise to the so-called nucleation potential, which requires a higher overpotential than that needed to reduce Ag^+^ ions. The shift in the second cathodic peak toward more positive potentials is attributed to the absence of newly formed nuclei; instead, previously formed nuclei continue to grow [13].

The electrochemical response of SPCE–Ag compared to bare SPCE, obtained by CV using a solution of 5 mM of K_3_[Fe(CN)_6_] and 0.1 M of KCl in Milli-Q water, is shown in Figure 5b. When comparing the CV profiles of SPCE–Ag and SPCE, a decrease in the difference in peak potential (ΔE) is observed upon Ag deposition on the SPCE (ΔE_(SPCE–Ag)_ = 210 mV vs. ΔE_(SPCE)_ = 140 mV), along with a narrowing of the working potential window. The increase in electrochemical reversibility can be attributed to improved electrode conductivity resulting from the incorporation of a metallic phase on the surface, which enables more reversible redox processes than those observed for the bare electrode.

**Figure 6:**
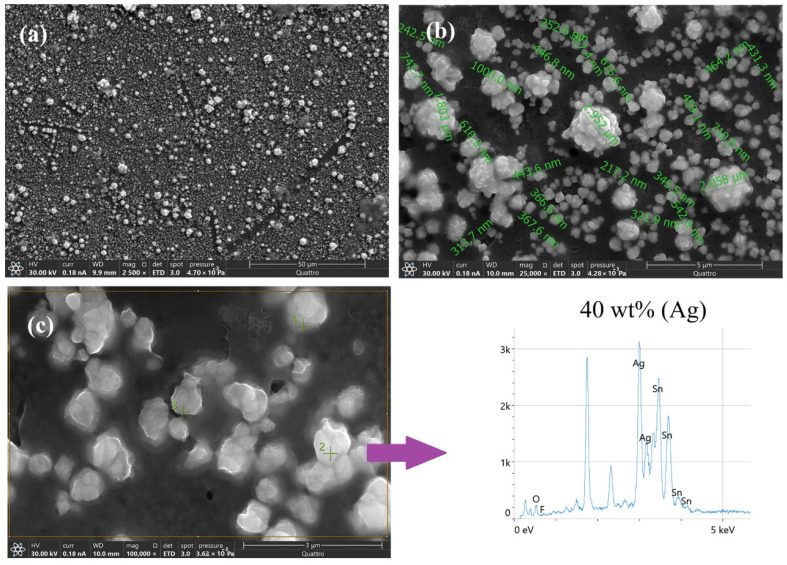
(**a**) FESEM micrographs of Ag obtained by two CV cycles. (**b**) Average particle sizes of the obtained Ag particles. (**c**) EDS analysis of SPCE–Ag.

The morphology of these particles was analyzed using FESEM, as shown in Figure 6a,b at different magnifications. As observed in Figure 6a, synthesis using two CV cycles enables the formation of Ag, as evidenced by the white structures in the micrograph, which are homogeneously distributed and exposed on the electrode surface, thereby facilitating subsequent modification with the proposed antibodies. At higher magnification (Figure 6b), the former silver structures exhibit a wide size distribution, ranging from nanometer-sized particles to larger micrometer-scale structures, which are formed through the agglomeration of smaller particles.

Additionally, energy-dispersive X-ray spectroscopy (EDS) was used to analyze the surface elemental composition of the deposits (Figure 6c). The obtained spectra clearly reveal the presence of Ag, which accounts for approximately 40 wt% of the total mass on the electrode surface, thereby confirming successful electrochemical modification.

**Figure 7:**
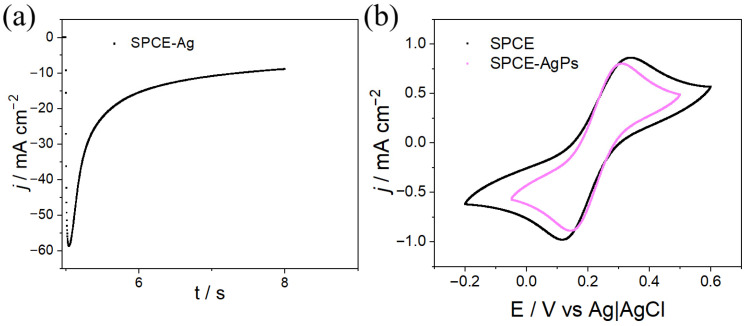
(**a**) Current density–time (j/t) transients for the electrosynthesis of SPCE–Ag using a 3 s pulse (2.3 M KSCN + 0.05 M Ag_2_SO_4_ in Milli-Q water). (**b**) Electrochemical response of SPCE and SPCE–Ag obtained by CA (5 mM K_3_[Fe(CN)_6_] + 0.1 M KCl in Milli-Q water, v = 100 mV s^−1^).

The Ag deposition by CA was optimized at −0.61 V, corresponding to the potential at which the first cathodic peak appears in the CV. Potential pulses at different times were applied, revealing that a 3 s pulse improved particle distribution and homogeneity. As shown in Figure 7a, the application of the 3 s pulse leads to an abrupt decrease in the current signal, associated with the formation of the electrical double layer at the electrode interface and the generation of Ag nuclei, a process resembling instantaneous nucleation. This phenomenon occurs rapidly; subsequently, a decrease in current is observed, attributed to the growth of the previously generated nuclei.

The electrochemical responses evaluated under the same conditions as those in Figure 7b, using CV, show, similarly to the previous case, a decrease in the separation of the redox peaks for electrodes on which silver was deposited for 3 s (ΔE = 160 mV), which also indicates an increase in the reversibility of the modified electrode.

**Figure 8:**
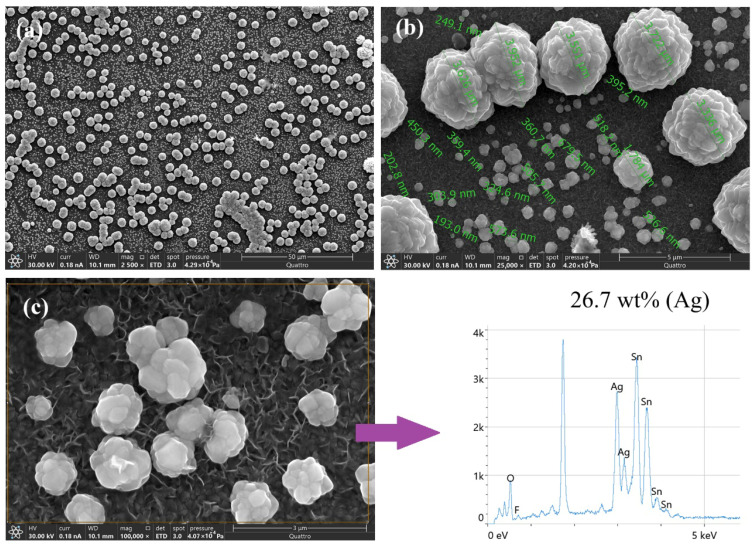
(**a**) FESEM micrographs of Ag obtained by CA. (**b**) Average particle sizes of the obtained Ag particles. (**c**) EDS analysis of SPCE–Ag.

Although the electrochemical reversibility is lower when electrosynthesis is performed by CA, the FESEM images of these deposits reveal a more uniform surface distribution and greater homogeneity in silver particle sizes (Figure 8a,b). EDS analysis of the areas shows higher Ag intensity, indicating greater metal content in these regions, corresponding to 26.7 wt% Ag. These results corroborate the successful electrochemical deposition of Ag using short potential pulses (Figure 8c).

### 3.3. Electrochemical Preparation of SPCE–PPy–AgPs

**Figure 9:**
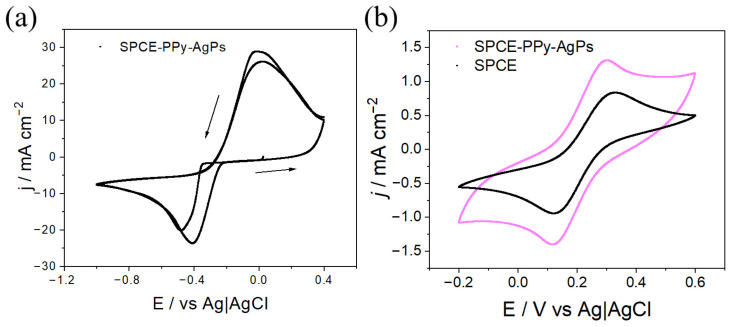
(**a**) Cyclic voltammogram for the electrosynthesis of SPCE–PPy–Ag (0.05 M Ag_2_SO_4_ + 2.3 M KSCN in Milli-Q water, v = 100 mV s^−1^). (**b**) Voltammetric profiles of SPCE and SPCE–PPy–Ag obtained by two CV cycles (5 mM K_3_[Fe(CN)_6_] + 0.1 M KCl in Milli-Q water, v = 100 mV s^−1^).

After studying Ag electrodeposition on SPCE, the electrosynthesis of Ag on PPy-modified SPCEs was investigated using the previously determined parameters. It can be observed that when the synthesis is carried out by CV (Figure 9a), both reduction peaks shift toward more positive potentials compared to those observed for bare SPCE. This behavior can be explained by the presence of nitrogen atoms in the PPy chains, which interact with Ag^+^ ions and adsorb them on the electrode surface, where they are subsequently reduced to metallic Ag [14,15,16]. The same phenomenon observed for SPCE–Ag is detected, with the appearance of a first reduction peak at −0.50 V, corresponding to the formation of the initial nucleation centers, and a second peak at −0.41 V, associated with the shift in the signal due to the growth of nuclei formed during the previous cycle [13,17].

Subsequently, the electrochemical response of SPCE–PPy–Ag obtained by CV was evaluated using a solution of 5 mM K_3_[Fe(CN)_6_] and 0.1 M KCl in Milli-Q water. When comparing the voltammetric profiles of SPCE–Ag and SPCE–PPy–Ag (Figure 9b), a decrease in the separation of the redox peaks is observed upon silver deposition on the electrode (ΔE_(SPCE–Ag)_ = 210 mV vs. ΔE_(SPCE–PPy–Ag)_ = 183 mV). This behavior is attributed to the increase in the electrochemical reversibility resulting from Ag deposition, as well as to an increase in current density, associated with the presence of PPy. The porous structure of PPy increases the effective electrode area, thereby improving the electrochemical response [18,19].

To achieve a homogeneous distribution of Ag particles on the PPy surface, CA was performed at −0.50 V, a potential value determined from CV (the first cathodic peak). The synthesis was carried out using potential pulses ranging from 3 to 5 s, yielding results similar to those previously observed in Figure 7a.

**Figure 10:**
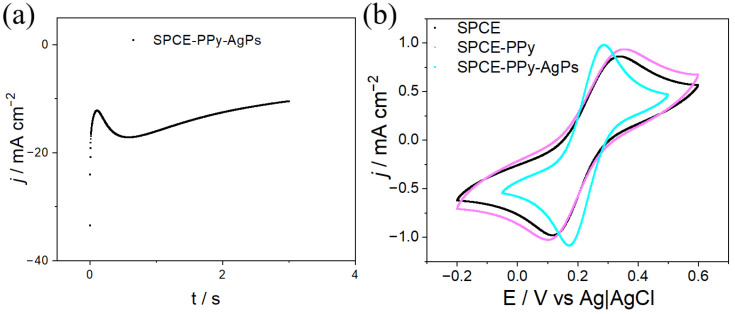
(**a**) Current density–time (j/t) transients for the synthesis of AgPs on SPCE–PPy by applying −0.5 V for 3 s (2.3 M KSCN + 0.05 M Ag_2_SO_4_ in Milli-Q water). (**b**) Electrochemical response at each electrode modification step (5 mM K_3_[Fe(CN)_6_] + 0.1 M KCl in Milli-Q water, v = 100 mV s^−1^).

Figure 10a shows an abrupt decrease in the current signal during the first second, corresponding to the formation of the electrical double layer at the electrode interface and the generation of Ag nuclei, a process resembling instantaneous nucleation. Subsequently, a further decrease in the signal is observed, which is attributed to the growth of the previously generated nucleation centers.

The CVs of SPCE–PPy–Ag electrodes synthesized by CA were compared with those of SPCE and SPCE–PPy and are shown in Figure 10b. From this comparison, it is observed that the deposition of AgPs on the PPy surface with a 3 s pulse leads to a shift in the peak potential difference (ΔE) from 255 mV to 117 mV for SPCE–PPy–Ag, SPCE, and SPCE–PPy, respectively. This behavior demonstrates increased electrochemical reversibility. In addition, an increase in the magnitude of the oxidation and reduction peaks is observed, attributed to the enhanced effective surface area and higher conductivity resulting from the incorporation of AgPs.

**Figure 11:**
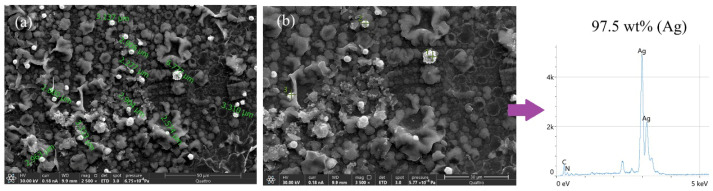
(**a**) FESEM micrographs of SPCE–PPy–Ag obtained with a 3 s pulse. (**b**) EDS analysis of SPCE-PPy–Ag.

The FESEM characterization of SPCE–PPy–AgPs is presented in Figure 11a. The obtained micrographs show that the Ag structures deposited on the PPy films retain a predominantly globular morphology, with a higher degree of dispersion compared to AgPs synthesized in the absence of PPy. This improved dispersion is expected to favor a more efficient surface modification with the Ki-67 antibody. In addition, the homogeneous distribution of AgPs reduces particle agglomeration and yields more uniform particle sizes than those observed without PPy, leading to lower electrode-to-electrode response variability and enhanced system reproducibility. This behavior can be attributed to the ability to control, through CA, the number of effective nucleation sites from which AgPs subsequently grow.

EDS analyses of SPCE–PPy–AgPs samples synthesized with a 3 s pulse (Figure 11b) revealed pronounced peaks in the spectra, with elemental weight percentages of 97.5% Ag, 1.5% C, and 0.7% N, attributed to AgPs and PPy, respectively. These results confirm that the white structures formed on the PPy surface correspond to metallic Ag and demonstrate that electrochemical synthesis by CA enables the successful deposition of Ag on PPy films.

### 3.4. Immobilization of Ki-67 Antibodies

The immobilization of the Ki-67 antibody was performed directly on the modified SPCE–PPy–AgPs electrode. For this purpose, a 20 μL aliquot of a Ki-67 solution (10 μg mL^−1^) prepared in PBS buffer (pH 7.3) was drop-cast onto the electrode surface and incubated for 1 h at room temperature. This procedure promotes interactions between the nitrogen-sites in the amino groups of the antibody and the AgPs, leading to the formation of Ag–N bonds [20,21,22], as schematically illustrated in Equation (4).

(4) $$R-H_{2}N:+Ag^{0}\to R-H_{2}N-Ag^{0}$$

Although the exact mechanism of antibody immobilization on silver particles has not been fully elucidated, research points to electrostatic interactions and possibly a coordinate covalent bond between the lone pair of electrons on nitrogen and an empty orbital on silver [20].

**Figure 12:**
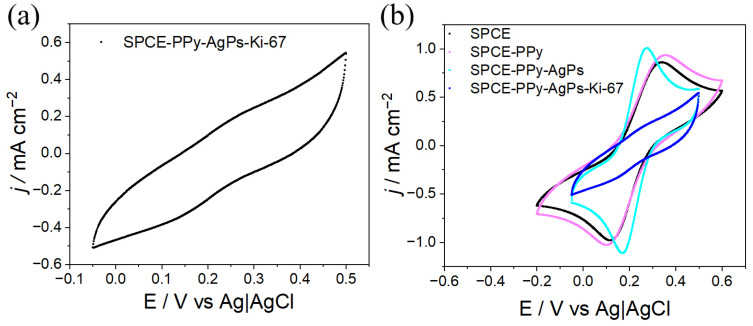
(**a**) Electrochemical response of SPCE–PPy–AgPs–Ki-67 (5 mM K_3_[Fe(CN)_6_] + 0.1 M KCl in Milli-Q water, v = 100 mV s^−1^). (**b**) Voltammetric responses corresponding to each electrode modification step (5 mM K_3_[Fe(CN)_6_] + 0.1 M KCl in Milli-Q water, v = 100 mV s^−1^).

The CV responses of the SPCE–PPy–AgPs–Ki67 system were evaluated using the ferricyanide/ferrocyanide redox couple, as shown in Figure 12a. After antibody immobilization, the characteristic redox signals almost completely disappeared, attributed to the proteinaceous, electrically insulating nature of antibodies. In addition, their relatively large molecular size creates a physical barrier that impedes the diffusion of the redox couple to the electrode surface, leading to pronounced passivation.

When analyzing reproducibility and electrode-to-electrode variability at this stage of the modification (n = 4), the relative standard deviation (RSD%) is 3.18% and 4.43% for the oxidation and reduction peaks, respectively, indicating that the antibody-modified electrode is highly reproducible.

The comparison of the CV responses at different stages of the surface modification process (Figure 12b) more clearly reveals the progressive blocking of the conductive surface upon the incorporation of the Ki-67 antibody. This behavior is consistent with that reported in the literature for electrochemical systems functionalized with antibodies or other biological recognition elements [23,24,25,26,27] and constitutes a reliable indicator of successful antibody immobilization on the electrode surface.

### 3.5. Evaluation of the Stability of SPCE–PPy–AgPs and SPCE–PPy–AgPs–Ki-67

The stability of the modified electrodes, with and without the Ki-67 antibody immobilized on gold nanoparticles, was evaluated by measuring the redox response using an external ferrocyanide redox probe after 24 h.

**Figure 13:**
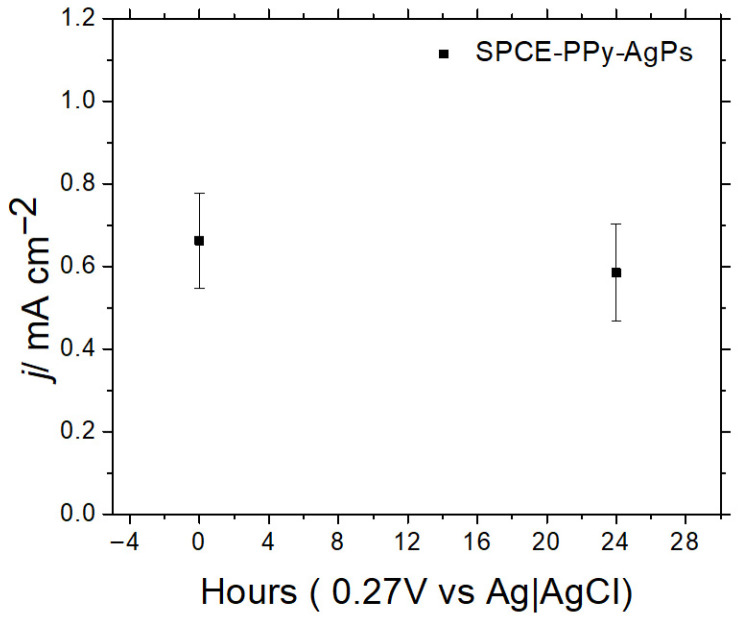
Variation in current density for SPCE-PPy-AgPs after 24 h (n = 5).

**Figure 14:**
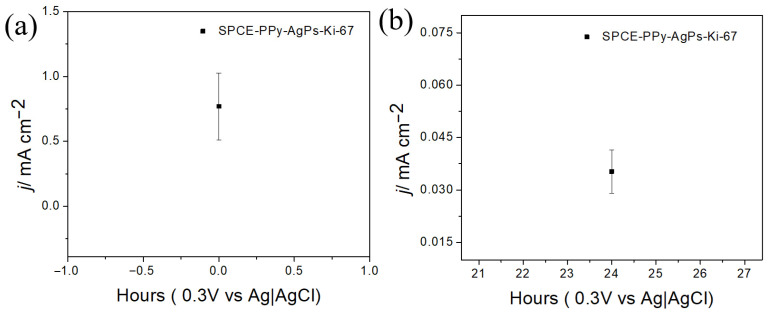
Variation in current density for SPCE-PPy-AgPs-Ki-67 (**a**) blank (**b**) after 24 h (n = 5).

As shown in Figure 13, when the antibody is not immobilized on the surface, the loss of electrochemical response decreases by 11.6% compared to the bare electrode. However, when the antibody is immobilized on the electrode surface (Figure 14), a significant 95.4% decrease in the electrochemical response is observed after 24 h.

In this regard, complex and bulky systems, such as antibodies, likely require stable environments to maintain acceptable reproducibility in biosensor construction. Nevertheless, it is encouraging to observe that, up to the modification step prior to antibody immobilization, surface stability is maintained. Indeed, it is highly feasible that future biosensor fabrication strategies will involve antibody immobilization at the time of use, while allowing the electrodes to be stored at the previous modification stage without significant loss of electrochemical signal.

### 3.6. Interference Study for SPCE-PPy-AgPs-Ki-67

**Figure 15:**
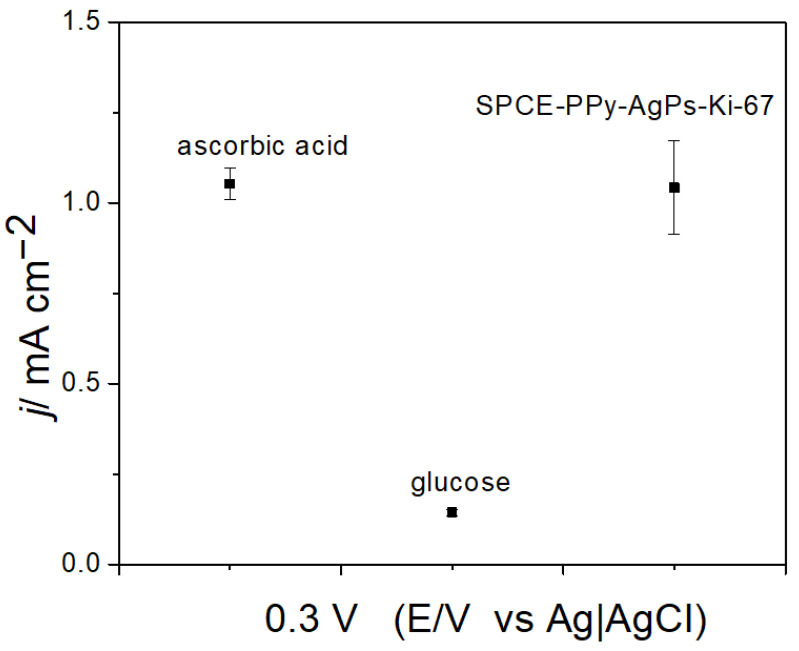
Comparison of the current density variability of SPCE-PPy-AgPs-Ki-67 in the presence of two interferents (ascorbic acid and glucose; n = 3).

Two common interfering species present in blood that could affect future biosensor measurements, namely glucose and ascorbic acid, were evaluated (Figure 15). For this evaluation, the same concentrations of both interferents were used, and the response of the external ferrocene redox couple was measured in their presence.

As shown in Figure 15, the presence of glucose produced a greater effect on the redox response of the external redox couple than ascorbic acid. In fact, the variability between the SPCE-PPy-AgPs-Ki-67 system and the signal obtained in the presence of ascorbic acid was only about 0.95%. However, the presence of glucose passivated the electrode surface, decreasing the redox couple response by 86.3%, thereby making glucose an interferent that should be removed from the sample matrix before biosensor measurements.

The observed differences in behavior between glucose and ascorbic acid can be attributed to surface adsorption and electrode passivation. Glucose can adsorb onto the PPy and silver nanoparticle surfaces through interactions with hydroxyl groups, forming a partial blocking layer that hinders electron transfer between the electrode and the ferrocene redox probe. Additionally, glucose oxidation products may contribute to surface fouling, further decreasing the electrochemical response. In contrast, ascorbic acid does not form a passivating layer under these conditions and remains highly soluble, resulting in a negligible effect on the electrochemical signal. Therefore, glucose acts as a significant interferent, whereas ascorbic acid does not significantly affect the electrochemical response of the SPCE-PPy-AgPs-Ki-67 system.

## 4. Conclusions

In this study, a hybrid electrochemical platform based on SPCEs modified with PPy and AgPs was designed and comprehensively characterized as a functional interface for antibody immobilization, with potential applications in advanced electrochemical biosensors. The rational integration of a nanoporous conducting polymer with Ag particles enabled the development of a highly active, conductive, and biocompatible surface that simultaneously functions as an electrochemical transducer and an efficient support for biological recognition elements.

The electrosynthesis of PPy on SPCEs, monitored by cyclic voltammetry and chronoamperometry, yielded homogeneous, stable, and electrochemically active films. Optimization of the experimental parameters demonstrated that applying a 1.3 V potential pulse for 10 s yields complete, reproducible coatings while preventing polymer overoxidation. Voltammetric analyses confirmed a significant enhancement in charge-storage capacity and electroactive surface area compared with the bare electrode. SEM characterization revealed a rough, nodular, and highly porous morphology typical of electropolymerized conducting polymers, which is particularly favorable for the subsequent incorporation of metallic species.

Silver electrodeposition was investigated and optimized on both SPCE and SPCE–PPy surfaces, revealing that the conducting polymer favorably influences the nucleation and growth of metallic particles. The deposition by chronoamperometry at potentials near the first cathodic peak enabled the formation of AgPs with a more homogeneous spatial distribution and a narrower particle-size dispersion than those obtained by cyclic voltammetry. The interaction between Ag^+^ ions and nitrogen sites in the PPy backbone promoted the surface retention of silver species and shifted the reduction potentials to more positive values, thereby facilitating stable nucleation. Consequently, SPCE–PPy–AgPs electrodes exhibited enhanced electrochemical reversibility, reduced redox peak separation, and increased current density, consistent with improved conductivity and an enlarged effective surface area.

Morphological analysis by SEM and compositional characterization by EDS confirmed the predominant presence of metallic Ag^0^ on the PPy matrix, with a well-dispersed distribution and a marked reduction in particle agglomeration. This hybrid architecture is particularly advantageous, as it not only enhances the electrode’s electrochemical performance but also provides a suitable platform for biomolecular immobilization, thereby improving reproducibility and reducing electrode-to-electrode variability.

Finally, direct immobilization of the Ki-67 antibody onto the SPCE–PPy–AgPs surface was achieved via interactions between the antibody’s amino groups and the Ag nanoparticles. The near-complete suppression of the ferricyanide/ferrocyanide redox signals following biofunctionalization evidenced a pronounced passivation effect, attributable to both the protein’s insulating nature and its molecular size, thereby confirming effective electrode functionalization. This behavior, widely reported for immunosensor platforms, validates the proposed strategy and highlights the potential of the developed system for immunorecognition-based electrochemical sensing.

The stability and analytical performance of the SPCE-PPy-AgPs-Ki-67 system were systematically evaluated, demonstrating that electrode modification prior to antibody immobilization yields a stable, reproducible platform. While incorporating the Ki-67 antibody significantly decreases the electrochemical response after 24 h, likely due to the formation of a blocking biological layer, the antibody-free modified electrode exhibits good stability, supporting its suitability for storage at intermediate fabrication stages.

Furthermore, the interference study revealed that glucose markedly affects the electrochemical response, causing substantial signal suppression due to electrode surface passivation and hindered electron transfer. In contrast, ascorbic acid showed a negligible effect on the signal, indicating that not all common blood components compromise sensor performance to the same extent.

These findings highlight the importance of controlling both surface functionalization and sample composition in the development of reliable biosensors. The results suggest that future strategies should focus on minimizing nonspecific surface blocking and implementing sample pretreatment steps to remove critical interferents such as glucose, while preserving the stability and functionality of the sensing platform.

Overall, these results demonstrate that the combination of PPy and AgPs on SPCEs constitutes a robust, reproducible, and versatile approach for constructing biofunctionalized electrochemical interfaces, providing a solid foundation for the future development of electrochemical biosensors targeting clinically relevant biomarkers such as Ki-67, with promising applications in diagnostic and clinical monitoring.

## Figures and Tables

**Table 1:** Charges of the doping/undoping processes obtained from the cycle 6 response of the SPCE and SPCE-PPy in monomer-free solutions.

Samples	Q_dop_ [C]	Q_und_ [C]	Q_dop_/Q_und_
SPCE	0.00328	0.00329	0.99696
SPCE-PPy	0.02872	0.02834	1.01413

## Data Availability

The original contributions presented in this study are included in the article. Further inquiries can be directed to the corresponding authors.

## Abbreviations

The following abbreviations are used in this manuscript:

SPCE	Screen-printed carbon electrode
PPy	Polypyrrole
AgPs	Silver particles
CA	Chronoamperometry
CV	Cyclic voltammetry
EDS	Energy-dispersive X-ray spectroscopy
